# What Factors Affect Farmers’ Levels of Domestic Waste Sorting Behavior? A Case Study from Shaanxi Province, China

**DOI:** 10.3390/ijerph191912141

**Published:** 2022-09-25

**Authors:** Yalin Yuan, Minyue Xu, Hanxin Chen

**Affiliations:** College of Economics and Management, Northwest A&F University, Yangling 712100, China

**Keywords:** domestic waste management, descriptive norms, injunctive norms, ordered logit model, rural areas

## Abstract

Waste sorting is a key element for solving the current predicament of rural waste management. In the pilot areas of China, farmers’ domestic waste sorting behavior (DWSB) varies significantly, whereas there are few studies exploring the mechanism of its formation. To fill this research gap, this study constructs a research model of the internal logic of farmers’ waste sorting levels (i.e., no sorting; sorting recyclable waste; sorting recyclable and kitchen waste; and sorting recyclable, kitchen, harmful, and other waste) by considering circumstantial constraints (social norms in external factors) and psychological behavioral antecedents (personal norms and group identity in internal factors). Based on pilot survey data from farmers in Shaanxi Province, China, the results of the ordered logit model indicate that social norms and personal norms were the most significant predictors of the level of DWSB, while group identity was found to have no significant influence. Furthermore, the results of the grouping regression analysis showed that personal norms had a positive moderating effect on the relationship between social norms and farmers’ DWSB. Therefore, a more positive social atmosphere, better education, and personal environmental moral responsibility for domestic waste sorting should be established to enhance their levels of waste sorting behavior.

## 1. Introduction

The massive generation of domestic waste and corresponding environmental pollution are major threats to the sustainable development of society. Particularly in rural China, driven by modern lifestyle and consumption habits, the total generation of domestic waste exploded from 90.5 million tons in 2014 to 164.7 million tons in 2018 [1]. As a result of long-term, substantial informal dumping practices, such as open-air and ditch dumping, large amounts of rural domestic waste have accumulated, causing critical adverse consequences and posing serious challenges and threats to the sustainable development of society and human health. For instance, a large proportion of land resources are now utilized for waste storage and disposal, underground water, soil, and air are contaminated, and many sites have become breeding spaces for flies, mosquitoes, and bacteria [2]. In addition, the COVID-19 pandemic continues to contribute to the increase in domestic waste generation and exacerbates the waste problem due to social distancing measures, whereby those staying at home engage in panic buying and intensify the consumption of single-use products [3,4,5].

Domestic waste sorting at the source is a key determinant of the success of the entire waste management process because it helps minimize landfill usage, lowers waste disposal charges, and reduces raw material usage through recycling programs [2]. In recent years, the Chinese government has successively promulgated various waste management strategies to promote the reduction, reuse, and recycling of rural domestic waste, thus optimizing the recycling system and building an effective recycling platform. For example, in 2017, the Ministry of Housing and Urban-Rural Development of China launched pilot programs on a source separation in 100 counties. However, due to the limitations of residents’ insufficient consciousness regarding environmental responsibility and normative management measures, the assessment of these pilot counties has had a minimal effect, and there are significant differences in the level of farmers’ domestic waste sorting behavior (DWSB) [6]. Waste management strategies based on waste sorting and recycling will only be successful if widespread public support is achieved [7]. Therefore, understanding the predictors of farmers’ levels of waste sorting behavior is important to enhance the source separation of domestic waste.

In recent years, waste sorting behavior has been intensively analyzed. A review of the literature reveals tools that can be used to promote waste sorting behavior, including external and internal factors. Among external factors, national environmental control policies, laws and regulations, and infrastructure and incentive policies are the main factors that influence DWSB. Through regulations or policies, the government and organizations can establish a waste disposal policy system that limits rural people’s free-riding behavior caused by the positive externalities of waste treatment [8]. Additionally, providing facilities and other convenient factors can directly promote the separation of domestic waste at the source [9,10]. Moreover, DWSB can be regarded as a pro-social and altruistic behavior involving positive externalities, which means that one sacrifices one’s needs to achieve the well-being of others [11]. According to the theory of welfare economics, intervention measures such as incentive instruments in the form of taxes, subsidies, and deposit refunds could benefit and encourage individuals to consistently engage in DWSB [12]. Among internal factors, attitude, willingness, and cognition all impact DWSB. Attitude, a variable of the theory of planned behavior toward waste sorting, is the main predictor of willingness to undertake domestic waste sorting, thus allowing the prediction of an individual’s actual DWSB [13,14]. Studies have shown that individuals who are more knowledgeable about domestic waste sorting can understand the adverse consequences of waste disposal without sorting and the positive outcomes of waste sorting, thus promoting their active participation in waste sorting and recycling activities [2,15,16]. Wang et al. [11] argued that farmers’ behavior is the result of both internal and external factors. It is noteworthy that most studies in this area have focused on external or internal factors on DWSB, whereas research concentrating comprehensively on the impact of both factors on different levels of DWSB is relatively limited.

Although researchers have found that laws and regulations are good predictors of increased waste sorting, monitoring people to achieve sustained waste sorting has proven difficult [17]. In the social psychology literature, it is suggested that the presence of norms, that is, informal rules requiring one to act in a given way in a given situation, may provide a pivotal impetus for people to remain conscious of environmental behavioral outcomes [18]. In addition, Wang and Zhang [19] found that compared with formal institutions, residents are more susceptible to social norms (SNs) as informal systems in their social environment. This may be a formal system without systematic consideration of the village situation caused by the lack of value principles that match village ethical concepts [20]. Among the various internal factors, personal norms (PNs) are considered a more internal and autonomous motivation for pro-environmental behavior [17,21]. Meanwhile, group identity (GI) has been rooted in Chinese society under the prolonged influence of Confucian and collectivist cultures and has had an essential influence on farmers’ behavior [6]. Tang et al. [6] found that farmers automatically classified other farmers to select their own group membership and paid more attention to the evaluation and recognition of members within the group. In addition, Kashima et al. [22] suggested that GI corresponds to the most internalized and meaningful source of motivation for environmental behavior. Personal intrinsic motivation inspires residents’ pro-environmental behavior, and they can acquire praise from others by sorting domestic waste to seek a sense of belonging and identity.

In this context, is there an association between external factors (SNs), internal factors (PNs and GI), and DWSB levels? First, as an external factor that reflects the common characteristics and behaviors of residents in similar ways, are SNs a pivotal impetus for them to implement an informal system? Second, since residents are eager to obtain positive evaluations and approval from others through their actions, from an intrinsic motivation perspective, might PNs and GI stimulate their pro-environmental behavior?

To enhance research on the levels of farmers’ sorting behavior, SNs were treated as an external factor in this research. At the same time, the effects of the internal factors, PNs and GI, were examined. Overall, considering the psychological behavioral antecedents and circumstantial constraints among farmers, this study addressed the associations among SNs, PNs, GI, and different levels of DWSB by including both external and internal factors. Meanwhile, to better comprehend the formation process of the different levels of farmers’ sorting behavior, the intrinsic logic and path between internal and external factors were analyzed. Finally, an empirical test was conducted using field survey data from pilot areas of domestic waste sorting in rural China.

The contributions of this study are as follows. First, this is one of the few studies that simultaneously analyze different levels of waste sorting behaviors (i.e., no sorting; sorting recyclable waste; sorting recyclable and kitchen waste; and sorting recyclable, kitchen, harmful, and other waste) and considers the logic of the interaction between external (SNs) and internal factors (PNs and GI). Second, considering the actual unsatisfactory domestic waste sorting situation in contemporary rural China and the scarce evidence in this area, this study is conducive to addressing the current practical waste sorting dilemma and provides directions for rural local governments and other similar areas to reconsider policies to encourage farmers to participate in waste sorting programs.

This paper is organized into six sections, including the introduction. Section 2 reviews the literature on the external (SNs) and internal (PNs and GI) determinants of pro-environmental behavior and presents four hypotheses. Section 3 focuses on the research methodology given the details of the sample, study scope, data collection, and measures used in this study. Section 4 and Section 5 highlight the results of the data analyses and discuss the findings, respectively. Section 6 presents the conclusions, implications, and limitations of the study.

## 2. Literature Review and Hypotheses

### 2.1. Social Norms

Adherence to SNs is crucial in modern society [23]. SNs are defined as a group of informal rules and standards that regulate the behavior of society members [24]. Such informal rules and standards often rely on the salience of two typical social norms: descriptive and injunctive [19,24]. Descriptive norms (SN_1_) are the typical behaviors of most people in the reference group, whereas injunctive norms (SN_2_) represent behaviors people (dis)approve of within a reference group [19,24,25]. That is, rather than simply informing one’s actions, these norms enjoin them through the promise of social sanctions [19,24].

Theories of conformity suggest that behavior is often shaped by people’s understanding of what others do. In this case, what others do is referred to as SN_1_, signaling the prevalence of a specific behavior [26]. Studies of SN_1_ and pro-environmental behavior have extensively examined the actions of others. For example, de Groot et al. [24] explained that littering is significantly more likely to occur in a setting in which litter is already present than in a clean setting. Although people know that littering is not right, they tend to act in accordance with clear behavioral norms. SN_2_ affects behavior based on a person’s beliefs about what others think they should or should not do. Accordingly, in this study, we assume that farmers’ DWSB is more easily triggered by the attitudes of the people around them or by external constraints. Considering the above, the following hypothesis is proposed.

**H1.**  *There is a positive relationship between SNs and the level of DWSB.*

### 2.2. Personal Norms

PNs, which are often regarded as a strong motivator of pro-environmental behavior, refer to the sense of a moral obligation to do the right thing [27]. That is, while social norms are rules and standards that have no force of law and are understood and followed by members of a group [24], PNs are rules or standards that govern one’s own behavior [27]. Indeed, previous studies have revealed that the stronger one’s PNs toward pro-environmental behavior, the more their behavior is related to this norm [28,29]. Accordingly, individuals with more moral obligations or responsibilities to protect the environment may be more actively involved in waste separation activities.

Obuobi et al. [30] claimed that PNs are internalized as a social norm. However, when an individual has stronger moral beliefs about a certain topic, the level of social validation that they receive from their surroundings should have less impact on them [31]. In situations where individuals might perceive that other people are not engaging in the desired behavior, stronger PNs may result in more of the desired behavior [32]. Therefore, PNs originate from individual conscious reasoning and contemplation regardless of social expectations [30]. Based on the above discussion, the following two hypotheses are proposed.

**H2.**  *There is a positive relationship between PNs and the level of DWSB*.

**H3.**  *PNs play a moderating role between SNs and the level of DWSB*.

### 2.3. Group Identity

According to the theory of social identity, GI can be defined as an individual’s decision to identify with a specific social group [33]. Many studies have suggested that GI is a positive determinant of an individual’s pro-environmental behavior. For example, Visschers et al. [34] indicated that GI is positively associated with an individual’s food sorting behavior. Rees and Bamberg [35] noted that GI is a direct predictor of collective climate behavior. Lin et al. [36] found that farmers with a higher degree of GI are more likely to participate in village ecological governance. Accordingly, in this study, it is posited that farmers will be more willing to participate in domestic waste sorting if they have a sense of belonging and identity with the village and that residents’ actual waste sorting behavior is more easily triggered when their sense of GI is strong. Thus, we propose the following hypothesis:

**H4.**  *There is a positive relationship between GI and the level of DWSB*.

**Figure 1:**
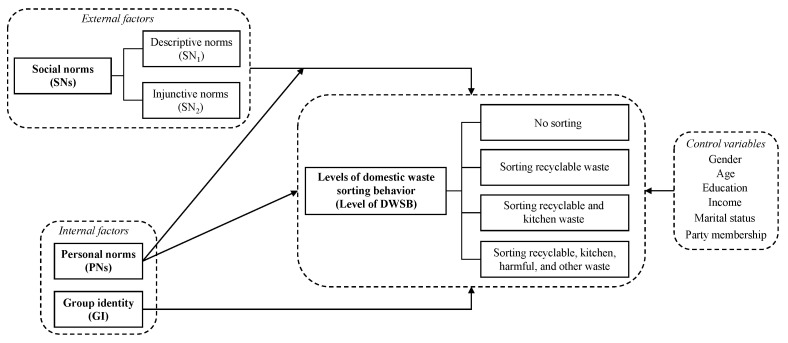
Research model.

The research model presented in Figure 1 summarizes the four hypotheses.

## 3. Data and Methodology

### 3.1. The Sample

A questionnaire survey was conducted in Shaanxi Province, China. Shaanxi Province, located in the hinterland of northwest China, is important as a birthplace of Chinese civilization with thousands of years of agricultural history. It has a population of more than 161.8 million, approximately 41.87% of whom live in rural areas [37]. Dali County, located in central Shaanxi Province, was one of the 100 pilot waste sorting counties in China in 2017. The local government has taken a series of measures to ensure the effective implementation of the waste sorting program. For example, all households have been equipped with four types of waste cans (recyclable, kitchen, harmful, and other waste), every village has built a landfill, each town has constructed waste compression transfer stations, and 2100 cleaners have been hired to service the towns and villages [38]. Furthermore, Dali County is in the process of building domestic waste incineration–hot spot co-generation project to realize the full incineration of domestic waste and realize the goal of zero landfills, which is expected to commence operations in 2021. Additionally, Dali’s economic scale, economic development level, and status of waste management are similar to those in most pilot counties, such as Shihe, Tonghua, Zhangzi, and Ningyuan. Thus, Dali County is a suitably representative research object to understand the level of farmers’ DWSB.

The survey was based on face-to-face interviews conducted between July and August 2019 with trained graduate students. Prior to this, a pre-survey with 60 resident questionnaires was conducted and reviewed in June 2019 to refine the preliminary questionnaire. Finally, considering the different rural environments and levels of economic development, 287 households were approached from Xiahan town in the west, Xuzhuang town in the center, and Chaoyi town in the east of Dali County, which was selected using random stratified sampling methods. Of these, 25 were deleted because of incomplete information, abnormal values, or repeated value problems. The final valid sample size was 262, with an effective response rate of 91.3%. Informed consent was obtained from all participants before they entered the study.

### 3.2. Measurements of the Main Variables

The measurement items used in the questionnaires were adapted from the existing valid scales, waste sorting standards in the investigated areas, and farmers’ waste disposal habits. For the explained variable, we measured the DWSB score on a scale from 0 = *no sorting* to 3 = *sorting recyclable, kitchen, harmful, and other waste*. Respondents’ perceptions of each of the three determinants—SNs (SN_1_ and SN_2_), PNs, and GI—were scored on a 5-point Likert scale ranging from 1 = *strongly disagree* to 5 = *strongly agree*.

**Figure 2:**
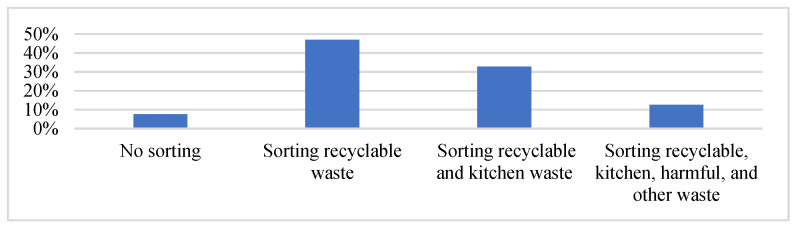
Distribution of the four categories of domestic waste management.

*Explained variables:* To measure farmers’ DWSB levels, the scales combined local separation rules (four categories: recyclable, kitchen, harmful, and other wastes) and farmers’ domestic waste disposal habits (two categories: recyclable and other wastes). We divided the level of farmers’ DWSB into four categories: no sorting; sorting recyclable waste; sorting recyclable and kitchen waste; and sorting recyclable, kitchen, harmful, and other waste. Figure 2 shows the distribution of the four DWSB categories. Farmers tend to sort part of their domestic waste: 32.8% said they sort recyclable and kitchen waste, while 46% stated that they only sort recyclable waste always or frequently. However, 7.6% never sorted their waste, and only 12.6% managed the required four categories (i.e., recyclable, kitchen, harmful, and other waste).

**Figure 3:**
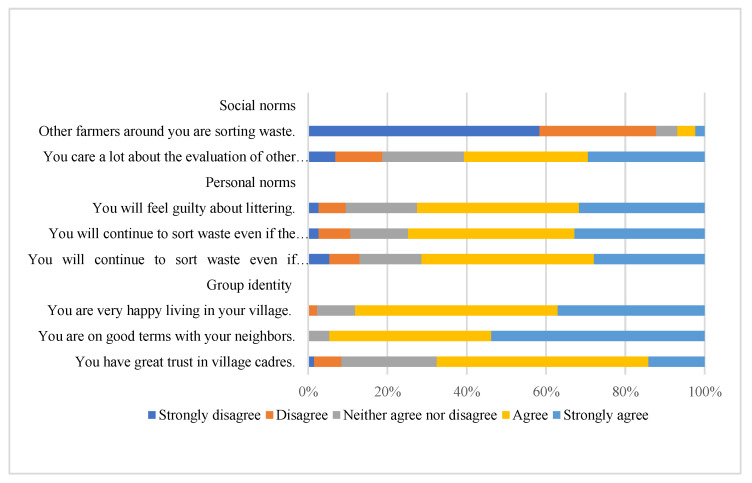
Responses to social norms, personal norms, and group identity.

*Explanatory variables:* SNs were measured using SN_1_ and SN_2_ [39], which were “Other farmers around you are sorting waste” and “You care a lot about the evaluations of other farmers”, respectively. Personal norms were measured using three items from Wang et al. [11]. These items aimed to reveal the respondents’ sense of responsibility for collective and environmental protection. “You will feel guilty about littering”, “You will continue to sort waste even if the improvement to the village environment is not obvious”, and “You will continue to sort waste even if the village does not give out free waste bags”. To measure GI, three items were adapted from Tang et al. [6]: “You are happy living in your village”, “You are on good terms with your neighbors”, and “You have great trust in village cadres”. Figure 3 summarizes the responses to the questions on SNs, PNs, and GI, whose distributions show wide scatter. The results of the correlation analysis indicate collinearity of the three items of PNs and three items of GI. To improve the stability of the model, this study employed principal component analysis to tease apart these two factors and extract comprehensive indicators using SPSS 19.0 software (IBM SPSS Statistics, USA). The results show that the KMO values are 0.602 and 0.587, respectively, and both the spherical degree test *p*-values are 0.000, which indicates that the data are suitable for factor analysis [40].

*Control variables:* Demographic characteristics included basic information, such as age, gender, marital status, educational background, and income. Previous studies have shown that respondents’ age, gender, marital status, party membership, education level, and gross annual household income influence individual pro-environmental behaviors [41,42]. Therefore, these variables were considered control variables in this study. As Table 1 shows, about 50% of respondents in the sample are male; the average age of respondents was about 50, indicating that the main participants in waste management were middle-aged farmers; the average education level of 8.26 years indicates that residents have on average completed the nine years of compulsory education; the average gross annual income was approximately 10,243.7 RMB. The collected data were similar to those from the *2019 Statistical Yearbook of Shaanxi Province* [37].

### 3.3. Estimation Method

The present study examines the level of DWSB expressed as ordered data. Traditional linear regression regards variables as cardinalities, which cannot satisfy the nature of discrete and ordered data [43]. The most well-known standard ordered response models are the ordered logit and probit logit models [43]. Compared to the probit logit model, the ordered logit model is a cumulative distribution function and does not require continuous variables [39]. Considering the above analysis, we applied the ordered logit model for the estimation. To introduce this model, we define an unobserved variable as $y_{i}^{*}$, which is a discrete index representing the level of DWSB. However, to simplify the interpretation of our results, we write

(1) $$y_{i}^{*}=\beta x_{i}+\varepsilon$$

where $x_{i}$ is a vector of explanatory variables, β is a vector of parameters, and ε is the random error.

Dividing $y_{i}^{*}$ into J ordinal categories

(2) $$y_{i}=m\;\mathrm{if}\;\tau_{n-1}\leq y_{i}^{*}\leq \tau_{n}\;\mathrm{for}\;n=1\;\mathrm{to}\;J$$

where the cut-points $\tau_{1}$ through $\tau_{J-1}$ are estimated. In the present study, the possible responses of the level of DWSB are: 0 = “no sorting”; 1 = “sorting recyclable waste”; 2 = “sorting recyclable and kitchen waste”; and 3 = “sorting recyclable, kitchen, harmful and other waste”. The observed response categories are tied to unobserved variables as follows:

(3) $$y_{i}=\left\{ \begin{array}{ll} 0=\mathrm{no sorting,} & \mathrm{if}-\infty \leq y_{i}^{*}\leq \tau_{1},\\ 1=\mathrm{sorting recyclable waste,} & \mathrm{if}\;\tau_{1}\leq y_{i}^{*}\leq \tau_{2},\\ 2=\mathrm{sorting recyclable and kitchen waste,} & \mathrm{if}\;\tau_{2}\leq y_{i}^{*}\leq \tau_{3},\\ 3=\mathrm{sorting recyclable, kitchen, harmful, and other waste,} & \mathrm{if}\;\tau_{3}\leq y_{i}^{*}\leq +\infty . \end{array}\right.$$

Hence, the cumulative probability of $y_{i}$ can be expressed as

(4) $$Pr\left(y_{i}=n|x\right)=Pr\left(\tau_{n-1}\leq y_{i}^{*}\leq \tau_{n}|x\right)$$

Substituting Equations (1)–(4) and adopting some algebra yields the standard formula, given as

(5) $$Pr\left(y_{i}=n|x\right)=F\left(\tau_{n}-\beta x_{i}\right)-F\left(\tau_{n-1}-\beta x_{i}\right)$$

where *F* denotes the cumulative distribution of ε. Note that for $F\left(-\infty -\beta x_{i}\right)$, the second term on the right-hand side of Equation (4) drops out since $F\left(-\infty -\beta x_{i}\right)=0$ and for $y_{i}=3$, the first term in Equation (4) equals $F\left(+\infty -\beta x_{i}\right)$.

## 4. Results

### 4.1. Descriptive Analysis

Table 1 reports the descriptions of all variables in this study. Regarding the measurement items of SNs, the mean value of SN_1_ is 1.630, while the average of SN_2_ is close to 4, which indicates that respondents believed that their actions were subject to others’ thoughts—what they should or should not do rather than imitating others’ behaviors. Principal component analysis was then used to identify the principal component variables that comprehensively reflect the intimacy of PNs and GI (see Table 2).

### 4.2. The Effects of Social and Personal Norms and Group Identity on the Level of Domestic Waste Sorting Behavior

Collinear diagnosis of variables was performed before regression using Stata 16.0 software (StataCorp LLC, USA). The variance inflation factor (VIF) of all variables was less than 1.39, indicating that the possibility of multicollinearity between variables was low [44]. Furthermore, a baseline regression of the DWSB level influencing factors model was performed without considering the moderating effect mentioned in the theoretical analysis (Table 3), and the explanatory variables were gradually introduced to test the explanatory power of the models. Model 1 only includes two variables reflecting SN_1_ and SN_2_, Model 2 introduces two variables of PNs and GI into Model 1, and Model 3 introduces all the control variables into Model 2. The log-likelihood and pseudo *R*^2^ values increased from −299.389 to −285.992 and 0.030 to 0.073 for Models 1 and 3, respectively. The results showed that the explanatory power of the model increased gradually after introducing PNs, GI, and the control variables. The variables in Model 4 are the same as those in Model 3, except that the ordinary least squares (OLS) method, which considers consistency in the direction and significance of parameter estimation with an ordered logit model [45], was adopted to test robustness. The estimation results from Models 1 to 4 show that the significance of the variables and the positive and negative signs of the coefficients do not change, indicating that the estimation results are robust. The following analysis is primarily based on the estimated results of Model 3. Table 4 shows the results of the marginal effect, which intuitively identifies the influence of the main variables on the DWSB level.

The results show that SNs are positively associated with DWSB levels. Consequently, Hypothesis 1 is empirically supported. Specifically, SN_1_ and SN_2_ had a remarkably positive influence on the level of DWSB (significant at the 1% level). The marginal effect results show that with a one-level increase in SN_1_, the probabilities of disposing of waste without sorting and sorting only recyclables decreased by 2.3% and 5%, respectively, while the probabilities of sorting waste into three and four categories increased by 3.9% and 3.3%, respectively. Similarly, with a one-level increase in SN_2_, the probabilities of not sorting waste and sorting only recyclables decreased by 1.6% and 3.5%, respectively, whereas the probabilities of sorting waste into three and four categories increased by 2.8% and 2.4%, respectively.

The results also indicated that PNs had a statistically positive association with DWSB level (significant at the 1% level). Therefore, Hypothesis 2 was empirically supported. The results of the marginal effect suggest that with a one-level increase in PN, the probabilities of disposing of domestic waste without sorting and sorting only recyclables decrease by 2% and 4.3%, respectively, while the probability of sorting domestic waste into three and four categories increases by 3.3% and 2.9%, respectively. It should be noted that GI did not have a significant effect on DWSB; thus, Hypothesis 4 was rejected. This effect may be related to the significant loss of population in rural areas, which has fostered a reduced sense of village identity.

Meanwhile, both party membership and gross annual income had statistically positive associations with DWSB (significant at the 1% level), which is similar to the findings of previous studies demonstrating positive correlations with recycling [42,46]. Female respondents reported being more likely to engage in the aforementioned domestic waste sorting than males (significant at the 10% level). These findings are consistent with those reported by Johnson et al. [47]. The negative association between educational attainment and waste management behavior has also been previously reported by Meneses and Palacio [48]. As expected, the relationships between age and marital status on the level of DWSB were negative (significant at the 1% level).

### 4.3. Moderating Effect of Personal Norms

Considering that SNs may have different effects on the DWSB of farmers with different levels of PNs, the PN variable was divided into high and low levels to further investigate its moderating effects. Specifically, based on principal component analysis, this study extracted a common factor measuring the PN variable with a mean value of 0 and a standard deviation of 1. A PN less than or equal to 0 was defined as low PN and assigned a value of 0, while a value greater than 0 was defined as high PN and assigned a value of 1. In addition, because the moderating variable of PN is categorical and the explanatory variable of SNs is continuous, a grouping regression analysis method was adopted [49].

The results for Model 5 (Table 5) show that the effects of SN_1_ and SN_2_ on DWSB remained significantly positive, further demonstrating the robustness of Model 3. In Models 6 and 7, the coefficients of SN_1_ and SN_2_ affected DWSB through different levels of PN, which increased from 0.164 and 0.148 to 0.601 and 0.278, respectively, indicating that PN played a positive moderating role between SNs and DWSB levels. Thus, Hypothesis 3 is accepted.

## 5. Discussion

Rural domestic waste pollution has significantly harmed the sustainable development of rural areas in China. Considering the problems of landfill shortages, resource conservation, disease spread, and environmental pollution, domestic waste sorting has major potential benefits for effective waste disposal [2]. Consistent with previous studies [29,39], this study confirms that farmers with higher levels of SNs and PNs are more likely to engage in higher levels of domestic waste sorting. We also found that PNs play a moderating role between SNs and DWSB levels. However, GI had no effect on farmers’ DWSB levels, corroborating Tang et al. [6]. We expect that the results will contribute to efforts to engage farmers and induce their participation in rural domestic waste sorting programs while enhancing the source separation levels of farmers’ domestic waste in a sustainable development context.

The fundamental finding is that SN_1_ and SN_2_ play positive roles in DWSB. Faced with the problem of sorting domestic waste into several categories, farmers are willing to imitate others’ behavior. In other words, if individuals observe that more people in their social circle tend to separate domestic waste into four categories, they are more likely to take similar actions [39]. In addition to learning from others’ behavior, others’ perceptions of individuals’ recycling efforts are an important driver of pro-environmental behavior [44]. As farmers live in relatively closed conditions, others’ cognition and expectations based on mutual communication play a vital role in their behavioral intentions in rural areas of China. Hence, they actively sort domestic waste, perhaps because they attach importance to others’ evaluations of it and share the same beliefs.

PNs also contributed to DWSB levels. In other words, farmers sort domestic waste based on personal moral norms. In other words, farmers who understand the advantages of sorting waste and the disadvantages of unsorting waste actively participate in domestic waste sorting, which is strongly associated with their personal environmental awareness and sense of responsibility. This observation is consistent with the results of previous studies [39,50].

This study found that GI was not related to the sorting of domestic waste. Although many previous studies have found that GI is an important indicator of pro-environmental behavior [51,52], in this study, farmers did not seem to make decisions about sorting domestic waste into different categories based on their sense of belonging and identity with villages in rural China. A possible explanation for this result is that farmers’ sense of belonging to the village collective has been weakened by the loss of a substantial portion of the rural population. As the rural social structure weakens with the shift from an acquaintance society to a semi-acquaintance society, the influence of GI on the level of DWSB declines [6].

Moreover, PNs are not only directly associated with DWSB levels but also moderate the relationship between SNs and DWSB levels. Specifically, SNs could further facilitate DWSB by inculcating farmers with higher personal environmental awareness and a sense of responsibility. These results are similar to those of Dwyer et al. [32], who showed that SNs promote energy conservation behavior at different levels of PNs. Our findings further validate that the strength of farmers’ PNs is likely to influence their DWSB levels, as people are influenced by their surroundings or other farmers’ actions [31,53], thus indicating that the moderating effect of PNs is robust.

Nevertheless, the current study has some limitations that require further exploration. By being restricted to cross-sectional data for a particular year, the empirical analysis may suffer from problems of endogeneity and may not directly indicate a convincing causal relationship between these variables. Second, although this study reflects useful facts for Guanzhong and other similar waste sorting pilot areas, the comparatively small sample size and selected location might limit the generalization of our findings. Therefore, the replication of this study should be considered in other contexts to broaden its generalizability.

## 6. Conclusions and Implications

DWSB plays a vital role in effective waste management. However, farmers’ DWSB varies significantly across the pilot areas of China. For example, in the pilot areas of Shaanxi Province, 46% of respondents declared that they only sorted recyclable waste always or frequently, 32.8% stated that they sorted recyclable and kitchen waste, 12.6% managed the required four categories, and 7.6% did not sort waste at all. Therefore, the promotion of DWSB remains a significant issue for policymakers. External (SNs) and internal (PNs and GI) factors could be the main determinants of enhanced sorting behavior. However, to the best of our knowledge, few studies have analyzed all these tools concurrently. Furthermore, it is important to analyze whether these aspects contribute to DWSB, considering the current situation of these tools. Therefore, with reference to a representative survey conducted in the pilot areas of Shaanxi Province in 2019, this study aimed to analyze the impact of SNs, PNs, and GI on DWSB. The results showed that the two SN dimensions had the most significant positive effects on waste sorting levels. In other words, the behavioral influence of surrounding people and external constraints can significantly improve farmers’ participation in domestic waste sorting. However, GI tools did not significantly affect this behavior. Further analysis indicated that there were differences in SNs in promoting waste sorting with different levels of PNs.

The results indicate several policy directions for rural local governments and other similar areas to encourage farmers to improve DWSB. First, the government should reinforce the guidance and constraints of informal institutions such as SNs to enhance village culture, as the conduct and moral norms of farmers are reinforced through village committees and other grassroots organizations to improve SN_1_ and enhance farmers’ domestic waste sorting levels. It is also beneficial to consolidate SN_2_ and inspire farmers’ personal moral awareness by establishing informal social systems, such as village rules and folk contracts. Second, a variety of communication channels (television, newspapers, radio, magazines, and the Internet) can be adopted to disseminate waste sorting knowledge and further educate farmers on the value of correct waste sorting and the negative effects of disposing of inadequately sorted waste to cultivate PNs among farmers. Last but not least, punishing those who fail to adequately sort domestic waste can convince farmers to abide by village norms. It is also necessary to give full play to the exemplary role of the Communist Party of China members and women to actively boost farmers’ sorting of domestic waste.

With the advancement of rural domestic waste sorting in China, future research should further explore the levels of DWSB in a broader community and be subject to proper adjustments by considering differences in culture, beliefs, knowledge, region, and habits. Furthermore, the levels of DWSB in this study are reported by the interviewees, and there may be a deviation between the expression and the actual situation. The accuracy of levels of DWSB can be improved by combining the records of the researchers and instrument monitoring in future studies.

## Figures and Tables

**Table 1:** Description of variables.

Variables	Measurement Items and Coding	Mean	S.D.	Min	Max
Explained variables					
Level of DWSB	Domestic waste sorting behavior (DWSB) of respondents. No sorting = 0, Sorting recyclable waste = 1, Sorting recyclable and kitchen waste = 2, Sorting recyclable, kitchen, harmful, and other waste = 3	1.504	0.811	0	3
Explanatory variables					
SN_1_	Other farmers around you are sorting waste. Strongly disagree = 1, Disagree = 2, Neutral = 3, Agree = 4, Strongly agree = 5	1.630	0.495	1	5
SN_2_	You care a lot about the evaluation of other farmers. Strongly disagree = 1, Disagree = 2, Neutral = 3, Agree = 4, Strongly agree = 5	3.650	1.213	1	5
PNs	According to the principal component analysis	0.000	1.000	−4.000	1.401
GI	According to the principal component analysis	0.000	1.000	−3.334	1.454
Control variables					
Gender	Gender of respondents. Male = 0, Female = 1	0.490	0.501	0	1
Age	Age of respondents (years old)	50.300	16.575	13	78
Education	Education of respondents (years)	8.260	3.582	0	17
Gross annual income	Gross annual income of respondents’ household (Ten thousand RMB)	3.329	3.706	0	40
Marital status	Marital status of respondents. No = 0, Yes = 1	0.130	0.332	0	1
Party membership	Party membership of respondents. No = 0, Yes = 1	0.090	0.289	0	1

Note: The proportion of four levels of DWSB—no sorting; sorting recyclable waste; sorting recyclable and kitchen waste; sorting recyclable, kitchen, harmful, and other waste—are 7.6%, 46.9%, 32.8%, and 12.6%, respectively.

**Table 2:** Component score coefficient matrix of personal norms and group identity.

Items		Component Score Coefficient
For each statement, please indicate how much you agree or disagree: Strongly disagree = 1, Disagree = 2, Neutral = 3, Agree = 4, Strongly agree = 5.		
PNs	Personal norms	
1	You will feel guilty about littering.	0.501
2	You will continue to sort waste even if the improvement to the village environment is not obvious.	0.491
3	You will continue to sort waste even if village does not give out free waste bags.	0.389
GI	Group identity	
1	You are very happy living in your village.	0.474
2	You are on good terms with your neighbors.	0.445
3	You have great trust in village cadres.	0.368

**Table 3:** Regression results from social and personal norms and group identity on the level of DWSB.

Variables	Model 1 (Logit)		Model 2 (Logit)		Model 3 (Logit)		Model 4 (OLS)	
	Coef.	S.E.	Coef.	S.E.	Coef.	S.E.	Coef.	S.E.
SN_1_	0.321 ***	0.127	0.331 ***	0.126	0.335 ***	0.131	0.134 ***	0.050
SN_2_	0.321 ***	0.216	0.278 ***	0.104	0.237 ***	0.105	0.090 ***	0.043
PNs	—	—	0.284 ***	0.129	0.287 ***	0.133	0.116 ***	0.054
GI	—	—	0.040	0.125	−0.076	0.134	−0.038	0.057
Gender	—	—	—	—	−0.226 *	0.245	−0.090 *	0.096
Age	—	—	—	—	−0.281 ***	0.119	−0.112 ***	−0.112
Marital status	—	—	—	—	−1.095 ***	0.500	−0.437 ***	−0.437
Party membership	—	—	—	—	0.924 ***	0.287	0.365 ***	0.112
Education	—	—	—	—	−0.135 *	0.146	−0.050 *	0.055
Gross annual income	—	—	—	—	0.105 *	0.087	0.040 *	0.034
Log Likelihood	−299.389		−296.480		−285.992		—	
LR (P > chi^2^)	18.940 (0.000)		24.310 (0.000)		45.290 (0.000)		—	
Pseudo *R*^2^	0.030		0.039		0.073		—	
F (P > F)	—		—		7.330 (0.000)		5.850 (0.000)	
Observation	262							

Note: *** *p* < 0.01, * *p* < 0.1.

**Table 4:** Marginal effect of ordered logit model.

Variables	Sorting Level = 0	Sorting Level = 1	Sorting Level = 2	Sorting Level = 3
SN_1_	−0.023 *** (0.098)	−0.050 *** (0.193)	0.039 *** (0.153)	0.033 *** (0.134)
SN_2_	−0.016 *** (0.077)	−0.035 *** (0.155)	0.028 *** (0.122)	0.024 *** (0.108)
PNs	−0.020 *** (0.098)	−0.043 *** (0.196)	0.033 *** (0.156)	0.029 *** (0.135)
GI	0.005 (0.092)	0.011 (0.200)	−0.009 (0.157)	−0.008 (0.135)
Gender	0.015 * (0.169)	0.034 * (0.363)	−0.026 * (0.285)	−0.023 * (0.246)
Age	0.019 *** (0.088)	0.042 *** (0.174)	−0.033 *** (0.138)	−0.028 *** (0.122)
Marital status	0.074 *** (0.367)	0.163 *** (0.733)	−0.128 *** (0.582)	−0.109 *** (0.509)
Party membership	−0.063 *** (0.229)	−0.137 *** (0.410)	0.108 *** (0.331)	0.092 *** (0.298)
Education	0.009 * (0.101)	0.020 * (0.216)	−0.016 * (0.170)	−0.013 * (0.146)
Gross annual income	−0.007 * (0.006)	−0.016 * (0.129)	0.012 * (0.102)	0.010 * (0.088)

Note: *** *p* < 0.01, * *p* < 0.1; standard errors are reported in parentheses.

**Table 5:** Moderating effect of personal norms between social norms and the level of DWSB.

Variables	Model 5 (All the Samples)		Model 6 (Low PN)		Model 7 (High PN)	
	Coef.	S.E.	Coef.	S.E.	Coef.	S.E.
SN_1_	0.330 ***	0.131	0.164 *	0.192	0.601 ***	0.205
SN_2_	0.234 ***	0.105	0.148	0.330	0.278 **	0.278
PNs	0.267 ***	0.128	—	—	—	—
Control variables	Controlled					
Log Likelihood	−286.151		−149.264		−129.908	
LR (P > chi^2^)	44.790 (0.000)		14.400 (0.109)		33.600 (0.000)	
Pseudo *R*^2^	0.073		0.046		0.115	
Observation	262		139		123	

Note: *** *p* < 0.01, ** *p* < 0.05, * *p* < 0.1.

## Data Availability

The dataset used in the path analyses and the full questionnaire are available from the corresponding author upon request.
